# Infant Feeding in Health and Disease

**Published:** 1901-05

**Authors:** 


INFANT FEEDING IN HEALTH AND DISEASE. A modern book on all Methods of Feeding. For Students, Practitioners and Nurses. By LOUIS FISCHER, M. D., Attending Physician to the Children's Service of the New York German Poliklinik; Bacteriologist to St. Mark's Hospital; Professor of Diseases of Children in the New York School of Clinical Medicine; Attending Physician to the Children's Department of the West Side German Dispensary. Containing 52 illustrations, with 16 charts and tables, mostly original. 368 pages. Philadelphia: F. A. Davis Company. 1901. [Price, $1.50, net]

Dr. Fischer has written a little book which every physician who has anything to do with infant feeding will welcome. It is one of those rare medical works over which one is apt to grow extravagant in praise, containing as it does so much that one often desires to know and has no means of finding out.

The first chapters are devoted to the anatomy and physiology of the infantile digestive tract. Breast feeding and the clinical examination of breast-milk are fully considered. Then follow chapters on the various methods of substitute feeding. Part second deals especially with the feeding of sick children, including the feeding of the prematurely born, diphtheria intubation cases, and rectal feeding.

Analysis of the most common proprietary foods, not in the dry state, but as prepared for use, are given. This alone makes the volume worth its cost, for who has not desired to know what he was actually giving his little patients? Laboratory or modified milk is discussed at considerable length, the experience of Dr. Fischer in the use of this form of infant food being that of many, if not most who have tried it.

Altogether this is a most complete and practical treatise on infant feeding and one which deserves a ready and large sale.

